# Text Messages as a Reminder Aid and Educational Tool in Adults and Adolescents with Atopic Dermatitis: A Pilot Study

**DOI:** 10.1155/2010/894258

**Published:** 2010-09-01

**Authors:** Venessa Pena-Robichaux, Joseph C. Kvedar, Alice J. Watson

**Affiliations:** ^1^Research Department, Center for Connected Health, 25 New Chardon Street, Suite 400D, Boston MA 02114, USA; ^2^Dermatology Department, Massachusetts General Hospital, MA 02114, USA; ^3^Department of Dermatology, Harvard Medical School, MA 02115, USA

## Abstract

Optimal management of atopic dermatitis (AD) requires patients to adhere to self-care behaviors. Technologies, such as cell phones, have been widely adopted in the USA and have potential to reinforce positive health behaviors. We conducted a pilot study with 25 adolescents and adults age 14 years and older [mean 30.5 yrs, SD 13.4] with AD. Daily text messages (TMs) that provided medication reminders and AD education were sent for six weeks to participants. Our goals were to (1) measure changes in pre- and posttest scores in treatment adherence, self-care behaviors, disease severity, and quality of life and (2) assess the usability and satisfaction of the TM system. Significant improvements in treatment adherence, self-care behaviors, skin severity, and quality of life (*P* ≤ .001, .002, <.001, and .014, resp.) were noted postintervention. User feedback on the TM system was positive with 88% and 92% of participants reporting that the reminder TMs and educational TMs were helpful, respectively. In conclusion, study participants were receptive to using TMs as a reminder aid and educational tool. The positive trends observed are promising and lay the ground work for further studies needed to elucidate the full potential of this simple and cost-effective intervention.

## 1. Introduction

Atopic dermatitis (AD) is a common chronic skin disease, accounting for 30% of dermatologic consults in general practice and 10%–20% of dermatologists' referrals [1]. Adherence to self-care behaviors, such as taking medication regularly, amongst patients with AD tends to be poor [2–5]. Given the morbidity [6–11] and economic burden [6, 12, 13] associated with AD, it is essential that effective strategies are developed to support patients and promote self-care behaviors. 

Both reminders and educational interventions have a positive impact on treatment adherence [14–18]. However, these are often delivered in a labor-intensive, costly way making them difficult to sustain or scale. The cell phone is a ubiquitous simple technology that may prove to be vital in helping to address difficulties with treatment adherence. Approximately four out of five adolescents and 89% of adults in the USA own a mobile phone [19, 20], and 55% of consumers utilize text messaging [21]. Text message (TM) reminders have already been used effectively to remind patients to attend medical appointments [22–24] and apply daily sunscreen [25], implicating their potential use to improve other aspects of patient adherence. Expanding the scope of TM interventions to deliver educational information in addition to reminders may prove to be an effective and affordable way to scale adherence interventions. 

In this study, we evaluated the use of TMs to provide treatment adherence reminders and patient education to adolescents and adults with AD. The purpose of this pilot study was to investigate the effect of such an intervention on various parameters of treatment adherence and patient outcomes. In addition, the usability and satisfaction of the TM intervention was assessed.

## 2. Methods

### 2.1. Study Design

This 6-week pilot study was conducted at Massachusetts General Hospital (MGH) in Boston, MA, and was approved by the Partner's Healthcare human research committee. Research participants were asked to attend two study visits, one at the beginning and one at the end of the six-week study. During the first visit a trained research assistant performed a skin evaluation to assess the severity of participants' AD. After the skin evaluations participant were asked to answer questions regarding demographic information and their current AD treatment regimens. Participants then filled out surveys that evaluated the following: AD treatment adherence and utilization of AD self-care behaviors. During the 6 weeks following their initial visit, all study participants received a daily TM that: (1) reminded them to continue their current treatment for AD, or (2) provided them with educational information about AD.

At the end of the 6 weeks participants returned for their final visit to MGH, where they received a second skin evaluation and were asked to answer surveys. The surveys were identical to those at the beginning of the study except that an additional section was added, which asked participants their feedbacks about the TMs they received over the 6 weeks.

### 2.2. Sample

Potential research participants were recruited through advertisements placed in local newspapers and magazines as well as online (Craigslist, Facebook, http://www.clinicaltrials.partners.org) in order to reach a diverse population. They were prescreened over the phone and were eligible to enroll if they were 14 years of age or older, had a primary care physician, had a diagnosis of AD that was made by their primary care physician or a dermatologist, were currently on treatment for their AD (including over-the-counter regimens), and had a cellular phone capable of receiving TMs. Twenty-nine individuals who met these criteria were identified and invited to participate in the study. Of these, 27 participants attended the first study visit, were rescreened, and gave their consent to participate (1 parent provided assent for their 16-year-old child). Two participants were dropped from the study due to changing circumstances that made them ineligible to participate, resulting in a completion rate of 93% (25/27).

### 2.3. Description of Text Message System

**Figure 1:**
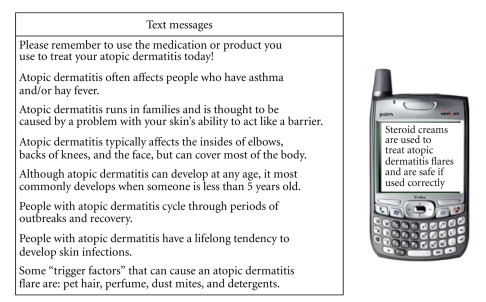
Examples of text messages sent to subjects.

Research participants received a daily TM throughout the 6 weeks of the study. The daily TM alternated between providing them with a reminder to continue to their AD treatment and giving them educational information about AD (reminders were given 3 times a week, and educational information was given 4 times a week) (Figure 1). Each participant was able to choose a time frame within which they would like to receive the TM (7 am–9 am or 4 pm–6 pm) and had the option of receiving an additional “fun” TM or “hook” in order to make receiving the TMs more enjoyable (participants chose between receiving the weather forecast, sports scores, or celebrity gossip). TMs were sent to participants using the TxtSignal website [26].

### 2.4. Outcome Measures

**Treatment Adherence**

Medication adherence was assessed at the first and last study visits in two ways. First, study participants were asked to fill out a 7-day recall calendar and mark the days during the last week when they were adherent to their AD treatment. Second, all participants were asked a multiple choice question in a survey that asked how often they forget to use their AD products/medication in the last week (responded “never”, “once or twice”, “three to five times”, or “six or more”). Pre- and postintervention self-reported adherence scores were compared, using a paired *t*-test.

**AD Self-Care Behaviors**

A survey listing 14 behaviors (Table 3) known to help improve or prevent AD flares was developed and administered to subjects at both study visits. Participants were asked whether they exhibited these behaviors “always”, “sometimes”, or “never”. An improvement in a self-care behavior was defined as a reported change in a positive direction (i.e., “never” to “sometimes”, or “sometimes” to “always”). Pre- and postintervention self-care behaviors were compared, using a paired *t*-test.

**Skin Severity**

AD severity was assessed using the SCORing Atopic Dermatitis index. A research assistant was trained on how to use this tool and performed all skin evaluations for the first and last research study visits. Pre- and postintervention SCORAD scores were compared, using a paired *t*-test.

**Quality of Life**

Quality of life was assessed using the Dermatology Quality of Life Index (DQLI) for those participants 17 years of age and older, and the Child Dermatology Quality of Life Index (CDQLI) for those 16 years of age and younger. Pre- and post-intervention DQLI/CDQLI scores were compared, using a paired *t*-test.

**Usability and Satisfaction of Text Message System**

Usability and satisfaction with the TMs was assessed via a survey administered at the final study visit. Participants were asked to rate the usefulness of the TMs, whether they would want to continue to use the TM system, if they would recommend the TM system to a friend, and if they experienced any problems with the TM system.

## 3. Results

### 3.1. Demographic Data

Research participants were adults and adolescents (14 years and older) currently on treatment for AD. Demographic information is presented in Table 1. The most common AD treatments being used by subjects at enrollment were OTC topical products (92%), followed by prescription topical medication (72%), oral prescription medication (12%), and oral OTC medication (8%).

### 3.2. Pre- and Postintervention Measures

Table 2 presents a summary of pre and post intervention measures.

**Treatment Adherence**

At enrollment, the majority of subjects reported that they sometimes forget to use their AD medications (92%) and often stopped their AD treatment when their skin symptoms improved (88%). In addition, one-third of participants (33.3%) reported that they stopped their AD treatment when their skin symptoms worsened. At the end of the 6-week intervention, 72% of participants reported improved adherence using both the 7-day recall calendar (preintervention mean 3.8 days [SD 2.4], postintervention mean 6.0 days [SD 1.7], *P* < .001) and the multiple-choice question.

**AD Self-Care Behaviors**

Over two-thirds of participants (68%) reported an improvement in the number of behaviors they were “always” performing (pre-intervention mean 3.6/14 [SD 2.3], postintervention mean 6.1/14 [SD 3.1], *P* = .002). In addition, 96% of participants reported an improvement in at least one self-care behavior after the TM intervention, and half of those reported improvement in at least 5 behaviors.

**Skin Severity**

Overall, there was a significant improvement in SCORAD scores (*P* < .001). 76% of participants had an improvement in their SCORAD score (mean change in score 7.89 [SD 4.5]), and of these participants, greater than one-third (36.8%) had an improvement ≥10 points.

**Quality of Life**

Quality of life as assessed by DQLI/CDQLI scores significantly improved overall (*P* = .014). 72% of participants had improvements in scores, with a mean change in score of 4.94 [SD 4.4]. Of those with an improvement in DQLI/CDQLI score, 44.4% had a change in score ≥5 and 11.1% had a change in score ≥10.

### 3.3. Usability and Satisfaction

On a scale from 1 to 10, participants rated the usefulness of the TM system a mean score of 7.1 [SD 2.4, min 2, max 10]. 88% of participants reported that they found the TM reminders helpful, and 92% reported that they found the educational texts helpful. If given a choice, 84% of participants said they would want to continue using the TM system, and 84% reported they would recommend the TM system to a friend. Over two-thirds of participants (72%) stated that they would be willing to pay a small monthly fee for this service. All participants stated that they were willing to use technology to manage their health care, and only 24% reported that they were worried about security issues of sending health information by email or phone. No problems regarding the TM system were reported.

## 4. Discussion

In this pilot study we demonstrated that text messages appear to be valuable both as a reminder aid and educational tool for subjects with AD. Subjects self-reported significant improvements in both medication adherence and self-care behaviors known to promote better clinical outcomes and quality of life. In addition, subjects expressed high levels of satisfaction with the TM intervention, with the majority willing to continue to use the service or recommend it to a friend. 

As reminder aids, TMs have been proven valuable for patients in many settings [22–24, 27] and help improve adherence to treatment [25, 28]. As such, the results from our study which illustrate improved adherence are not surprising. However, our study went a step further by integrating educational information into the TMs sent to subjects. This is something that to our knowledge has never been done, and we believe that providing patients with this additional educational information can serve as a stepping-stone for the improvements in self-care behaviors that were observed in this group. 

TMs can be used as an adjunct to conventional care for delivering patient education. Delivering education about AD through TMs may help patients to learn more about their skin disease and the behaviors that could help prevent flares. We believe educating patients in such a way could lead to improved attitudes towards treatment, positive changes in self-care behaviors, and improved clinical outcomes. 

Studies focusing on children with AD have found that education can have a positive impact on clinical outcomes in these patients [14–18]. However, the educational intervention used in these cases were parental workshops, which are often time consuming and inconvenient. TMs provide a cost-effective way to deliver short, concise segments of education over a longer period of time. Using TMs to deliver patient education requires no extra effort from the providers' side, as the system is automated. In addition, results from our study suggest that patients are willing and ready to begin integrating technology, such as TMs, into their care. In addition, online automated TM platforms are now beginning to support two-way communication, which indicates that there is much potential for growth with regard to the utilization of TMs for improving communication between patients and physicians.

## 5. Limitations

The results of this study must be considered in the context of the study design. As a pilot study with no control group, the data gathered can only point to possible trends that may occur in adolescent and adult patients with AD should a TM intervention be implemented into their care. In addition, we cannot say whether the effects observed are related to the intervention or the effect of study participation. Our sample size was also small with majority of participants being under the age of 30 and female. Although our study results are not immediately generalizable to all patients with AD in the US population, they do likely represent trends that would be seen in those young individuals who would be most likely to want to integrate TMs into the delivery of their healthcare. Thus, the results described here will be useful for informing the initial implementation of such tools. Adherence to treatment in this study was measured by self-report because more objective measures, such as electronic adherence monitors, are cumbersome and costly. Although using self-report to measure adherence is only moderately valid [29–32], an attempt to avoid discrepancies in self-report was made by having participants both answer a multiple choice adherence question and fill out a 7-day recall calendar. Finally, although the website used for sending the automated TMs could confirm that the TMs were sent to the study participants, there was no way of knowing whether or not the TMs were open and read by their recipients. However, because the feedback from the usability and satisfaction portion of the survey was primarily positive it is possible that most participants did utilize the TM service as intended.

## 6. Conclusion

This study illustrates that the use of TMs, a simple and inexpensive technology, may be effective as a reminder aid and educational tool in young patients with AD. The implications of the possible impact of this intervention on clinical outcomes in this patient population should be confirmed by a randomized controlled trial. Further research is also needed to explore the possibility of incorporating TMs into the management of patients with other chronic dermatologic diseases (acne, psoriasis, etc.). The relatively recent revolution of cellular phones and the Internet has paved a new pathway for patient-doctor communication. Physicians must begin to utilize this technology to facilitate better delivery of education and healthcare.

## Figures and Tables

**Table 1:** Demographic characteristics of study participants.

Characteristics	*n* = 25
Age, years (mean [SD])	30.5 [13.4]
Gender, % female	72.0
Ethnicity, %	
Caucasian (nonHispanic)	36.0
Black (nonHispanic)	28.0
Hispanic	4.0
Asian/Pacific Islander	12.0
Mixed ethnicity	20.0
Education, %	
High school or less	20.0
Some college	25.0
College graduate	55.0
Household income, % less than $50,000	68.0
Employment, %	
Full-time employed	24.0
Part-time employed	20.0
Student	48.0
Other*	8.0

*Other: Homemaker or Retired.

**Table 2:** Pre- and Postintervention statistics.

	Preintervention	Postintervention	*P*-Value
*Treatment adherence*			
Mean number of days/week of adherence to treatment [SD]	3.8 [2.4]	6.0 [1.7]	<.001

*AD maintenance Behaviors*			
Mean number of behaviors reported as “always” performing out of 14 [SD]	3.6 [2.3]	6.1 [3.1]	.002

*Skin severity *			
Mean SCORAD score	33.4 [8.9]	28.2 [7.7]	<.001

*Quality of life*			
Mean CDQLI/DQLI	7.8 [5.2]	5.0 [3.8]	0.014

**Table 3:** List of self-care behavior statements in survey.

Self-Care behavior statements*	
I try to avoid heat, sweating, and dry environments	
I avoid using soaps, detergents, cleaning products, and other chemicals that could irritate my skin	
I avoid wearing clothing made of wool and synthetic fibers	
I avoid exposure to cigarette smoke	
I dust frequently to reduce exposure to dust mites	
I try to find ways to decrease stress and anxiety	
I avoid long (greater than 10–15 minutes) and hot baths and showers	
I bathe with lukewarm water	
I use a mild soap or nonsoap cleaner to remove dirt when I bathe	
I apply moisturizer after I bathe	
I avoid using lotions with a high water and low oil content	
I control itching by using antihistamines	
I cut my fingernails short to try to avoid skin damage from scratching	
I use wet dressings to control my itching	

*Subjects asked to respond with “always”, “sometimes”, or “never”.

## References

[1] Rook AR, Savin JA, Wilkinson DS, Rook AR, Wilkinson DS, Ebling FJG, Champion RH, Burton JL (1986). The prevalence, incidence and ecology of diseases of the skin. *Textbook of Dermatology*.

[2] Zaghloul SS, Goodfield MJD (2004). Objective assessment of compliance with psoriasis treatment. *Archives of Dermatology*.

[3] Richards HL, Fortune DG, O’Sullivan TM, Main CJ, Griffiths CEM (1999). Patients with psoriasis and their compliance with medication. *Journal of the American Academy of Dermatology*.

[4] Brown KK, Rehmus WE, Kimball AB (2006). Determining the relative importance of patient motivations for nonadherence to topical corticosteroid therapy in psoriasis. *Journal of the American Academy of Dermatology*.

[5] Krejci-Manwaring J, Tusa MG, Carroll C (2007). Stealth monitoring of adherence to topical medication: adherence is very poor in children with atopicdermatitis. *Journal of the American Academy of Dermatology*.

[6] Feldman S, Behnam SM, Behnam SE (2002). Involving the patient: impact of inflammatory skin disease and patient-focused care. *Pediatric Allergy and Immunology*.

[7] Spergel JM, Paller AS (2003). Atopic dermatitis and the atopic march. *Journal of Allergy and Clinical Immunology*.

[8] Iikura Y, Naspitz CK, Mikawa H (1992). Prevention of asthma by ketotifen in infants with atopic dermatitis. *Annals of Allergy*.

[9] Warner JO (2001). A double-blinded, randomized, placebo-controlled trial of cetirizine in preventing the onset of asthma in children with atopic dermatitis: 18 months’ treatment and 18 months’ posttreatment follow-up. *Journal of Allergy and Clinical Immunology*.

[10] Kiebert G, Sorensen SV, Revicki D (2002). Atopic dermatitis is associated with a decrement in health-related quality of life. *International Journal of Dermatology*.

[11] Su JC, Kemp AS, Varigos GA, Nolan TM (1997). Atopic eczema: its impact on the family and financial cost. *Archives of Disease in Childhood*.

[12] Feldman SR, Evans C, Russell MW (2005). Systemic treatment for moderate to severe psoriasis: estimates of failure rates and direct medical costs in a north-eastern US managed care plan. *Journal of Dermatological Treatment*.

[13] Ellis CN, Drake LA, Prendergast MM (2002). Cost of atopic dermatitis and eczema in the United States. *Journal of the American Academy of Dermatology*.

[14] Grillo M, Gassner L, Marshman G, Dunn S, Hudson P (2006). Pediatric atopic eczema: the impact of an educational intervention. *Pediatric Dermatology*.

[15] Moore EJ, Williams A, Manias E, Varigos G, Donath S (2009). Eczema workshops reduce severity of childhood atopic eczema. *Australasian Journal of Dermatology*.

[16] Cork MJ, Britton J, Butler L, Young S, Murphy R, Keohane SG (2003). Comparison of parent knowledge, therapy utilization and severity of atopic eczema before and after explanation and demonstration of topical therapies by a specialist dermatology nurse. *British Journal of Dermatology*.

[17] Chavigny J-M, Adiceom F, Bernier C, Debons M, Stalder J-F (2002). Assessment of an educational program in an “atopic school”: pilot study in 40 patients. *Annales de Dermatologie et de Venereologie*.

[18] Broberg A, Kalimo K, Lindblad B, Swanbeck G (1990). Parental education in the treatment of childhood atopic eczema. *Acta Dermato-Venereologica*.

[19] Teenagers: A Generation Unplugged. http://www.ctia.org/advocacy/research/index.cfm/AID/11483.

[20] One in Five U.S. Adults Has No Landline. http://news.digitaltrends.com/news-article/16281/one-in-five-u-s-adults-has-no-landline.

[21] Vlingo Issues 'Consumer Text Messaging Habits' Report. http://www.reuters.com/article/pressRelease/idUS140196+21-May-2008+PRN20080521.

[22] Geraghty M, Glynn F, Amin M, Kinsella J (2008). Patient mobile telephone ’text’ reminder: a novel way to reduce non-attendance at the ENT out-patient clinic. *Journal of Laryngology and Otology*.

[23] Chen Z-W, Fang L-Z, Chen L-Y, Dai H-L (2008). Comparison of an SMS text messaging and phone reminder to improve attendance at a health promotion center: a randomized controlled trial. *Journal of Zhejiang University: Science B*.

[24] Leong KC, Chen WS, Leong KW (2006). The use of text messaging to improve attendance in primary care: a randomized controlled trial. *Family Practice*.

[25] Armstrong AW, Watson AJ, Makredes M, Frangos JE, Kimball AB, Kvedar JC (2009). Text-message reminders to improve sunscreen use: a randomized, controlled trial using electronic monitoring. *Archives of Dermatology*.

[26] Txtsignal http://www.txtsignal.com/.

[27] Hanauer DA, Wentzell K, Laffel N, Laffel LM (2009). Computerized automated reminder diabetes system (CARDS): e-mail and SMS cell phone text messaging reminders to support diabetes management. *Diabetes Technology and Therapeutics*.

[28] Strandbygaard U, Thomsen SF, Backer V (2010). A daily SMS reminder increases adherence to asthma treatment: a three-month follow-up study. *Respiratory Medicine*.

[29] Minzi OM, Naazneen AS (2008). Validation of self-report and hospital pill count using unannounced home pill count as methods for determination of adherence to antiretroviral therapy. *Tanzania Journal of Health Research*.

[30] Smith SR, Wahed AS, Kelley SS, Conjeevaram HS, Robuck PR, Fried MW (2007). Assessing the validity of self-reported medication adherence in hepatitis C treatment. *Annals of Pharmacotherapy*.

[31] Cohen JL, Mann DM, Wisnivesky JP (2009). Assessing the validity of self-reported medication adherence among inner-city asthmatic adults: the Medication Adherence Report Scale for Asthma. *Annals of Allergy, Asthma & Immunology*.

[32] Conde JF, Kaur M, Fleischer AB, Tusa MG, Camacho F, Feldman SR (2008). Adherence to clocortolone pivalate cream 0.1% in a pediatric population with atopic dermatitis. *Cutis*.

